# Supplementary material for: Improving realty management ability based on big data and artificial intelligence decision-making
Source: PLoS One. 2024 Aug 14;19(8):e0307043. doi: 10.1371/journal.pone.0307043 (PMC11324138; doi:10.1371/journal.pone.0307043)
Supplement: Attachment — Submitted filename: Reviewer Report_PONE-D-24-06746.docx [file pone.0307043.s001.docx]

**Reviewer Report _** **PONE-D-24-06746**

Thanks for giving me a chance to review this manuscript. This is an interesting topic. The author(s) tries to work significantly. This article proposes a knowledge-dependent data processing scheme (KDPS) to augment precise data analysis. However, still, some of the anomalies I found during the review process are addressed below, which may help further develop the study.

1. **Originality**

It can be improved

-	The author(s) should illustrate how this research differs from the literature in the field / or considered a contribution to the literature.

-	For the **practical study gap**, the author (s) should focus on the concrete details of the situation in the study context:

(a.	Where and when does the problem arise? b. Who does the problem affect? c. What attempts have been made to solve the problem?)

-	For **theoretical research**, the author (s) should re-think about the scientific, social, geographical, and/or historical background:

(a.	What is already known about the problem? b. Is the problem limited to a certain time period or geographical area? c. How has the problem been defined and debated in the scholarly literature?)

The abovementioned issues could help to improve the originality of this study and make it stronger for interested readers, researchers, and/or practitioners in the field.

- The author(s) need to illustrate how their work could contribute to the knowledge in the field and how their work differs from previously published studies

2. **Relationship to Literature**

It can be improved

- Section 2. Should be renamed to be “Literature Review”
- In the “literature review” section, the author(s) should review the main sub-themes in this study topic. This section is recommended to conclude how the reviewed literature is different from the current study and how this study could contribute to the research area.
- The “literature review” should represent a survey of scholarly sources on this study topic. It should provide an overview of current knowledge, allowing both readers and authors to identify relevant theories, methods, and gaps in the existing research that could later be applied to this research work. **I believe that the authors should improve their “literature review” section to reflect that.**

3. **Methodology**:

Yes, the method is appropriate. **However**, it could be improved as follows:

-	Usually, the research methodology section recommended to include:

1.	The type of research you conducted

2.	How you collected and analyzed your data

3.	Any tools or materials you used in the research

4.	How do you mitigate or avoid research biases

5.	Why did you choose these methods

Please make sure that the “methodology” section includes the above-mentioned elements

4. **Results**

- A more thematic and detailed representation of the findings should be followed

**General Comments:**

- Both “theoretical and practical implications” should be improved and linked to the findings of the current study. The implications should be presented in a separate section
- The author(s) should be clear about which implications reflect the practical perspective and which reflect the theoretical perspective
- “Limitations and future research” should be presented in a separate section

.

**Quality of Communication:**

-	There is a need for proofreading and professional editing.
