# Supplementary material for: Improving realty management ability based on big data and artificial intelligence decision-making
Source: PLoS One. 2024 Aug 14;19(8):e0307043. doi: 10.1371/journal.pone.0307043 (PMC11324138; doi:10.1371/journal.pone.0307043)
Supplement: Attachment — Submitted filename: PONE-D-24-06746_reviewer_comments.docx [file pone.0307043.s002.docx]

The manuscript presents a promising contribution to the field of real estate property management, particularly in terms of data-driven analysis and decision-making. However, to achieve its full potential, the study requires substantial revisions to enhance clarity, methodological rigor, and overall presentation. Addressing the outlined recommendations will significantly improve the quality and impact of the research.

The introduction (section 1) lacks clear focus and structure, resulting in a disjointed presentation of ideas. The article jumps between various concepts and technologies without providing a coherent narrative or logical progression of thought. To improve readability and comprehension, the introduction should be organized into distinct sections with clear headings and subheadings to delineate different topics.

The article briefly touches upon various concepts such as cloud computing, big data extraction, AI-based decision-making, and machine learning algorithms without delving into their theoretical underpinnings or practical applications in real estate management. To enhance the scholarly depth of the article, each concept should be discussed in greater detail, providing definitions, explanations, and relevant examples to elucidate their significance in the context of real estate management.

While the article makes several claims about the benefits and efficacy of incorporating AI, big data analytics, and machine learning in real estate management, it lacks empirical evidence or case studies to support these assertions. To strengthen the argument and credibility of the article, empirical validation through case studies, experiments, or real-world examples should be provided to demonstrate the practical utility and effectiveness of the proposed knowledge-dependent data processing scheme (KDPS).

The recommendations provided at the end of the introduction are vague and lack specificity. They mention the introduction of a knowledge-based processing scheme and the incorporation of knowledge learning without elaborating on concrete steps or strategies for implementation. To provide actionable recommendations, the article should offer clear guidelines, methodologies, or frameworks for designing and implementing the proposed KDPS.

In section 1 (Introduction), it’s better to clarify the whole structure of the paper, which can be easier for reader to understand what scenarios have been investigated or examined and what’s main contribution this paper makes. For example, this paper is organized as follows: section 2 present what and section 3 present what… It’s suggested to give a brief overview of entire paper in the introduction.

The article presents a comprehensive list of related works without synthesizing the key findings, trends, or gaps in the existing literature. Instead of merely listing each work, the article should critically analyze the contributions, methodologies, and limitations of previous studies to identify overarching themes or research gaps. Synthesizing the literature would provide readers with a clearer understanding of the current state of knowledge in the field and highlight areas for further investigation.

The article lacks critical evaluation of the methodologies and findings of the referenced works. Each cited study is described briefly without assessing the strengths, weaknesses, or implications of the research. A critical evaluation would involve discussing the methodological rigor, applicability of findings, and potential biases in the studies cited.

While the article provides a comprehensive list of related works, it does not adequately discuss the relevance of each study to the proposed knowledge-based processing scheme (KDPS). The article should clearly articulate how each referenced work informs the development or validation of the KDPS and how it contributes to addressing the identified research problem. Discussing the relevance of previous studies would strengthen the rationale for the proposed approach and demonstrate its novelty or innovation.

One significant limitation is the scheme's reliance on historical data, which may not adequately capture real-time market dynamics. To address this, the KDPS should incorporate real-time data feeds and implement mechanisms for timely updates to adapt to changing market circumstances.

The effectiveness of the KDPS heavily relies on the quality and relevance of the features selected for analysis. There is a need to conduct thorough feature engineering and validation to ensure that the chosen features accurately reflect market trends and dynamics.

Transparency and interpretability are crucial for building trust and understanding in AI-driven decision-making systems. The KDPS should provide clear visual representations of its decision-making processes to stakeholders, such as decision trees or decision borders, to enhance comprehension and trust.

Dealing with missing data is critical for developing robust data processing systems. The KDPS should implement robust strategies for handling missing values, such as imputation techniques, and conduct sensitivity analyses to evaluate the impact of different approaches on model performance.

Optimal hyperparameter values significantly impact the performance of machine learning models. The KDPS should conduct thorough hyperparameter tuning studies to identify the best configuration for its algorithms and ensure optimal performance across different scenarios.

Rigorous validation and evaluation processes are essential for assessing the KDPS's performance and reliability. Implementing techniques such as k-fold cross-validation can provide insights into the model's generalization performance and robustness.

Revise the manuscript to improve clarity by defining technical terms, providing detailed explanations of methodologies, and ensuring logical coherence between sections.

Provide a clear, step-by-step description of data processing and analysis techniques, including any software tools or algorithms utilized. Incorporate references to established methodologies to enhance transparency and reproducibility.

Conduct thorough validation of the proposed approach using benchmark datasets or simulated scenarios. Compare the performance of the proposed method with existing models through quantitative metrics and qualitative evaluation criteria.

Simplify complex concepts and minimize technical jargon to make the manuscript accessible to a broader audience. Use clear, concise language and structured formatting to improve readability and comprehension.

While the conclusion briefly mentions the benefits of the proposed scheme, such as improved processing rate and success rate, it does not delve into the practical implications for real estate management practitioners or stakeholders.

Discussing how the findings can inform decision-making processes, optimize resource allocation, or enhance overall operational efficiency in realty management would provide valuable insights for readers interested in applying the research in practical contexts.

While the conclusion provides a concise summary of the research findings, it could benefit from greater specificity, depth, and discussion of practical implications. By enhancing the methodological explanation, providing illustrative examples, and discussing practical implications, the conclusion can better convey the significance and applicability of the research to readers.
