# Supplementary material for: Improving realty management ability based on big data and artificial intelligence decision-making
Source: PLoS One. 2024 Aug 14;19(8):e0307043. doi: 10.1371/journal.pone.0307043 (PMC11324138; doi:10.1371/journal.pone.0307043)
Supplement: Attachment — Submitted filename: response to reviewers.docx [file pone.0307043.s003.docx]

1. We are unable to verify the source of the clip art images used in Figures 1 -5 in your submission.
Before we can proceed with your manuscript, we ask that you please clarify the following: Please explain where the authors obtained the clipart images included within Figures 1 - 5 in your submission. If you you are unable to show that the source of these images clearly permits CC BY 4.0 reuse, please either remove the figures or supply replacement figures that comply with the CC BY 4.0 license.
Please check copyright information on all replacement figures and update the in-text figure captions with complete source information.

If applicable, please specify in the figure caption text when a figure is similar but not identical to the original image used in the study, and is therefore for illustrative purposes only.

**Ans:**

According to the above comments, the figures are self-drawn. Clip art images are removed in the diagram also each and every part of the diagram is self-drawn using draw.io software. There is no copy right issues are occurred while publishing the figure. The redrawn figures are illustrated as follows.

Figure 1: Property Analysis

Figure 2 KDPS Model

Figure 3 Gross Data Validation for Amenities Checking

Figure 4 Improvements Recommendation through Verification

Figure 5 Recommendation Process Functions

**Reviewer #1:** Thanks for giving me a chance to review this manuscript. This is an interesting topic. The author(s) tries to work significantly. This article proposes a knowledge-dependent data processing scheme (KDPS) to augment precise data analysis. However, still, some of the anomalies I found during the review process are addressed, which may help further develop the study.

1. **Originality**

It can be improved

-	The author(s) should illustrate how this research differs from the literature in the field / or considered a contribution to the literature.

**Ans:**

It is recommended that the KDPS be improved by integrating real-time data streams from diverse sources, including property listings, market trends, and economic indicators. The constant integration of real-time data into the system enables the analytical and decision-making processes to be grounded in the most current information. Furthermore, it is recommended that the KDPS incorporate methods to facilitate prompt updates and model retraining, so enabling the system to swiftly adjust to evolving market conditions. One potential approach is to implement periodic or event-driven updates, wherein the knowledge base and decision rules are revised in accordance with the most recent data and market conditions. By overcoming this constraint, the KDPS may offer more precise and pertinent observations, empowering real estate experts to make well-informed choices that accurately represent the present condition of the market.

-	For the **practical study gap**, the author (s) should focus on the concrete details of the situation in the study context:

(a.	Where and when does the problem arise? b. Who does the problem affect? c. What attempts have been made to solve the problem?)

Ans:

The issue of enhancing real estate management proficiency emerges within the realm of real estate management, wherein the proficient and proficient management of property-related data holds paramount importance. Multiple parties, such as property owners, real estate agents, property managers, and tenants, are impacted by this matter. Efforts have been undertaken to address this issue by means of the advancement of diverse data processing methodologies and technology. One method is implementing knowledge-dependent data processing schemes (KDPS) to improve the management of real estate data by utilizing domain-specific knowledge and norms. In the processing and analysis of property-related data, these schemes employ many techniques including rule-based systems, expert systems, and ontology-based approaches to effectively collect and apply domain knowledge. Furthermore, researchers have also investigated advancements in fields such as machine learning and artificial intelligence in order to enhance the precision and effectiveness of real estate data processing.

-	For **theoretical research**, the author (s) should re-think about the scientific, social, geographical, and/or historical background:

(a.	What is already known about the problem? b. Is the problem limited to a certain time period or geographical area? c. How has the problem been defined and debated in the scholarly literature?)

The abovementioned issues could help to improve the originality of this study and make it stronger for interested readers, researchers, and/or practitioners in the field.

- The author(s) need to illustrate how their work could contribute to the knowledge in the field and how their work differs from previously published studies

Ans:

The primary objective of the knowledge-dependent data processing scheme (KDPS) is to optimize real estate management through the utilization of domain-specific knowledge and data analysis methodologies. Data acquisition and organization encompass the collection and arrangement of pertinent information from diverse sources, including property listings, market trends, and client preferences. The data is further subjected to processing and analysis utilizing knowledge-based rules and algorithms that integrate domain experience derived from specialists in the real estate industry. The insights that have been retrieved are utilized for the purpose of constructing prediction models and decision support systems. These systems are designed to aid in various tasks such as property assessment, investment analysis, and client targeting. By integrating data-driven insights with human experience, KDPS empowers real estate managers to make well-informed decisions, resulting in greater operational efficiency, optimized resource allocation, and improved customer satisfaction

The incorporation of domain-specific information and experience into the data analysis process is a limitation that is addressed by the knowledge-dependent data processing scheme (KDPS). The utilization of human expertise and decision-making rules allows the system to effectively manage limitations in data and surmount computational intricacies. The versatility of KDPS enables its application in several industries, effectively overcoming the challenge of limited generalizability through the integration of industry-specific knowledge and data sources.

2. **Relationship to Literature**

It can be improved

- Section 2. Should be renamed to be “Literature Review”
- In the “literature review” section, the author(s) should review the main sub-themes in this study topic. This section is recommended to conclude how the reviewed literature is different from the current study and how this study could contribute to the research area.
- The “literature review” should represent a survey of scholarly sources on this study topic. It should provide an overview of current knowledge, allowing both readers and authors to identify relevant theories, methods, and gaps in the existing research that could later be applied to this research work. **I believe that the authors should improve their “literature review” section to reflect that.**

Ans:

Section 2 renamed as literature review

Literature review discussing the various methods involved in the realty management process to improve the overall quality, in addition, the study used to identify the problems involved in the management system.

The detailed explanation for each work is described in section 2. During the study, each method, problem, weakness and strength has been described according to your comment.

3. **Methodology**:

Yes, the method is appropriate. **However**, it could be improved as follows:

-	Usually, the research methodology section recommended to include:

1.	The type of research you conducted

Ans:

The KDPS can be classified as a decision support system (DSS) or a knowledge-based system (KBS) research study. Decision support systems are information systems designed to assist decision-makers in complex decision-making scenarios by providing relevant information, data analysis, and decision models. Knowledge-based systems, on the other hand, are systems that capture and utilize domain-specific knowledge and expertise to solve problems or provide recommendations.

2.	How you collected and analyzed your data

Ans:

This study uses the <https://www.kaggle.com/datasets/arashnic/property-data> dataset information to analyze the system efficiency. The collected data is processed according to the KDPS system which helps to making the effective decision.

3. Any tools or materials you used in the research

Ans:

This study uses the clustering and decision support system utilized to analyze the collected information.

4. How do you mitigate or avoid research biases

Ans:

To mitigate or avoid research biases, the KDPS employs several strategies, such as involving diverse domain experts to extract knowledge and decision rules, ensuring transparency in data preprocessing and analysis techniques, and conducting rigorous validation processes using techniques like cross-validation and hold-out testing. Additionally, the KDPS should incorporate mechanisms for continuous monitoring and updating to adapt to changing market conditions and incorporate new data sources, reducing the risk of biases arising from outdated or limited data.

5.	Why did you choose these methods

Please make sure that the “methodology” section includes the above-mentioned elements

Ans:
The selection of the KDPS methodologies was based on their capacity to effectively combine the specialized knowledge of real estate professionals with data-driven analysis tools. The utilization of a hybrid method not only guarantees the attainment of precise and dependable decision-making through the utilization of human expertise, but also addresses the difficulties that may arise from data sources that are inadequate or biased. Furthermore, the decision-making procedures employed by KDPS exhibit interpretability and transparency, hence cultivating confidence and acceptability among stakeholders. Moreover, the system's adaptability and flexibility enable it to maintain relevance amidst changing market conditions and emerging information. The KDPS methods offer a comprehensive and robust solution for real estate decision-making by utilizing knowledge representation, data mining, and machine learning techniques. These approaches are specifically designed to cater to the special requirements of the real estate domain.

5. **Results**

- A more thematic and detailed representation of the findings should be followed

Ans:

Results and discussion section part improved according to the comments and highlighted parts are helps to understand the improvements in the study.

According to the results, the knowledge-dependent data processing scheme (KDPS) demonstrates superior performance compared to the other techniques, specifically SMMP, BIM+AVM, and BTPM, in many real estate management measures. KDPS has the maximum processing rate of 253, which is much greater than the other approaches. On the other hand, SMMP has the lowest processing rate of 59. The KDPS strategy has the largest percentage of cases where the process becomes stagnant or stuck, measuring 42.312%. This indicates that KDPS is more effective than the other techniques in identifying and handling sluggish situations. KDPS exhibits the shortest processing time, measuring 2.153 seconds, indicating its exceptional efficiency and quickness in managing realty activities. KDPS approach exhibits the highest success rate, as evidenced by its proportion of effective outcomes at 0.844 or 84.4%. This indicates that KDPS outperforms the other methods in generating successful results. The training ratio, which quantifies the number of repetitions or iterations needed per input, is observed to be the highest for KDPS at 111. This suggests that achieving ideal performance may necessitate further training or exposure to data. The findings indicate that the knowledge-dependent data processing scheme (KDPS) demonstrates efficacy and efficiency in the context of realty management tasks. It surpasses alternative methods in multiple metrics, such as processing rate, stagnancy detection, processing time, success rate, and training ratio.

**General Comments:**

- Both “theoretical and practical implications” should be improved and linked to the findings of the current study. The implications should be presented in a separate section.
- The author(s) should be clear about which implications reflect the practical perspective and which reflect the theoretical perspective

**Ans:**

**Implication of this study is included in the end of the results and discussions.**

**Implication of this study**

**Table 1 Highlights of Performance Assessment (Data Inputs)**

| **Metrics** | **SMMP** | **BIM+AVM** | **BTPM** | **KDPS** |
| --- | --- | --- | --- | --- |
| Processing Rate (Requirements/ Input) | 59 | 125 | 201 | 253 |
| Stagnancy Detection (%) | 10.6 | 23.19 | 31.67 | 42.312 |
| Processing Time (s) | 6.21 | 4.27 | 2.79 | 2.153 |
| Success Rate | 0.512 | 0.645 | 0.758 | 0.844 |
| Training Ratio (Repetitions/ Input) | 42 | 68 | 83 | 111 |

**Findings:** The proposed scheme improves the processing rate, stagnancy detection, success rate, and training ratio by 8.2%, 10.25%, 10.28%, and 7%, correspondingly. It decreases the processing time by 8.56% compared to the existing methods.

**Table 2 Highlights of Performance Assessment (Amenities)**

| **Metrics** | **SMMP** | **BIM+AVM** | **BTPM** | **KDPS** |
| --- | --- | --- | --- | --- |
| Processing Rate (Requirements/ Input) | 62 | 121 | 202 | 237 |
| Stagnancy Detection (%) | 10.72 | 23.34 | 32.22 | 42.848 |
| Processing Time (s) | 6.29 | 4.29 | 3.03 | 2.095 |
| Success Rate | 0.521 | 0.642 | 0.747 | 0.842 |
| Training Ratio (Repetitions/ Input) | 32 | 75 | 102 | 125 |

**Findings:** The proposed scheme improves the processing rate, stagnancy detection, success rate, and training ratio by 7.64%, 10.38%, 10.27%, and 7.38%, correspondingly. It decreases the processing time by 8.96% compared to the existing methods.

6. Conclusion

- “Limitations and future research” should be presented in a separate section

**Ans:**

**Future work is included in the conclusion section.**

Future research endeavors may prioritize the advancement of the KDPS through the investigation of sophisticated knowledge representation methodologies, such as deep learning-based approaches, in order to effectively capture intricate patterns and correlations inherent in the dataset. Furthermore, the integration of real-time data feeds and the establishment of mechanisms for ongoing learning and adaptation would enhance the ability of the KDPS to deliver decision support that is both fast and accurate in the face of dynamic real estate market conditions.

**Quality of Communication:**

-	There is a need for proofreading and professional editing.

**Ans:**

Article proofreading is done.

**Reviewer #2:**

1. The manuscript presents a promising contribution to the field of real estate property management, particularly in terms of data-driven analysis and decision-making. However, to achieve its full potential, the study requires substantial revisions to enhance clarity, methodological rigor, and overall presentation. Addressing the outlined recommendations will significantly improve the quality and impact of the research.

Ans:

**Thanks for understanding the concept and contribution of the work. Overall the methodology and presentations are increased according to the comments given by reviewer comments. Then the recommendations are applied to the article which improves the overall quality of the manuscript and research.**

2. The introduction (section 1) lacks clear focus and structure, resulting in a disjointed presentation of ideas. The article jumps between various concepts and technologies without providing a coherent narrative or logical progression of thought. To improve readability and comprehension, the introduction should be organized into distinct sections with clear headings and subheadings to delineate different topics.

Ans:

**According to the comment, introduction section divided into different sub section and contribution of the study is described as separate section. The overall and entire topics are covered in the manuscript which improves the presentation of the work.**

3. The article briefly touches upon various concepts such as cloud computing, big data extraction, AI-based decision-making, and machine learning algorithms without delving into their theoretical underpinnings or practical applications in real estate management. To enhance the scholarly depth of the article, each concept should be discussed in greater detail, providing definitions, explanations, and relevant examples to elucidate their significance in the context of real estate management.

Ans:

Cloud computing is a significant component of real estate management since it facilitates the provision of computer services via the internet. This technology enables the storing, processing, and analysis of data on remote servers. This technology enables the retrieval and examination of large amounts of organized and unorganized data produced from many sources such as property listings, market trends, and consumer behavior. Artificial intelligence (AI) can be used in the real estate industry to automate property valuations, optimize pricing tactics, and uncover investment opportunities. This is achieved by utilizing algorithms that can learn from data and generate predictions or suggestions. Machine learning algorithms, which are a subset of artificial intelligence (AI), demonstrate exceptional proficiency in identifying patterns and may be trained using past data to make predictions about future trends in the real estate market, anticipate property prices, and provide recommendations for suitable renovations or improvements to optimize returns. An example of this would be a machine learning model that has been trained using historical sales data. This model would be capable of analyzing various attributes of a property and its surrounding area in order to make an estimation of its prospective market worth. This would greatly assist real estate agents and investors in making well-informed judgments.

4. While the article makes several claims about the benefits and efficacy of incorporating AI, big data analytics, and machine learning in real estate management, it lacks empirical evidence or case studies to support these assertions. To strengthen the argument and credibility of the article, empirical validation through case studies, experiments, or real-world examples should be provided to demonstrate the practical utility and effectiveness of the proposed knowledge-dependent data processing scheme (KDPS).

Ans:

This study uses the Rental Properties Collaboration Dataset for evaluating the efficiency of the knowledge-dependent data processing scheme (KDPS). The gathered information is processed according to the KDPS steps and the efficiency of the system is evaluated using different metrics such as processing rate, stagnancy detection rate, success rate, processing time etc. The detailed illustration of the validation or experimentation discussed in section 4.

5. The recommendations provided at the end of the introduction are vague and lack specificity. They mention the introduction of a knowledge-based processing scheme and the incorporation of knowledge learning without elaborating on concrete steps or strategies for implementation. To provide actionable recommendations, the article should offer clear guidelines, methodologies, or frameworks for designing and implementing the proposed KDPS.

Ans:

The primary objective of the knowledge-dependent data processing scheme (KDPS) is to optimize real estate management through the utilization of domain-specific knowledge and data analysis methodologies. Data acquisition and organization encompass the collection and arrangement of pertinent information from diverse sources, including property listings, market trends, and client preferences. The data is further subjected to processing and analysis utilizing knowledge-based rules and algorithms that integrate domain experience derived from specialists in the real estate industry. The insights that have been retrieved are utilized for the purpose of constructing prediction models and decision support systems. These systems are designed to aid in various tasks such as property assessment, investment analysis, and client targeting. By integrating data-driven insights with human experience, KDPS empowers real estate managers to make well-informed decisions, resulting in greater operational efficiency, optimized resource allocation, and improved customer satisfaction.

6. In section 1 (Introduction), it’s better to clarify the whole structure of the paper, which can be easier for reader to understand what scenarios have been investigated or examined and what’s main contribution this paper makes. For example, this paper is organized as follows: section 2 present what and section 3 present what… It’s suggested to give a brief overview of entire paper in the introduction.

Ans:

The rest of the paper is organized as follows. Section 2 describes the various researchers opinions regarding the realty systems. Section 3 discusses the working process of Knowledge-Dependent Data Processing Scheme based realty management process and the efficiency of the system evaluated in section 4. Conclusion described in Section 5.

7. The article presents a comprehensive list of related works without synthesizing the key findings, trends, or gaps in the existing literature. Instead of merely listing each work, the article should critically analyze the contributions, methodologies, and limitations of previous studies to identify overarching themes or research gaps. Synthesizing the literature would provide readers with a clearer understanding of the current state of knowledge in the field and highlight areas for further investigation.

Ans:

The comprehensive study of the existing work is discussed in section 2. According to the comment, the limitation and research gap of the work is included which helps to improve the overall contribution of the work. The included contents are highlighted.

Incorporating domain-specific information and experience into the data analysis process is a limitation addressed by the knowledge-dependent data processing scheme (KDPS). The utilization of human expertise and decision-making rules allows the system to manage limitations in data and surmount computational intricacies effectively. The versatility of KDPS enables its application in several industries, effectively overcoming the challenge of limited generalizability through integrating industry-specific knowledge and data sources.

It is recommended that the KDPS be improved by integrating real-time data streams from diverse sources, including property listings, market trends, and economic indicators. The constant integration of real-time data into the system makes the analytical and decision-making processes grounded in the most current information. Furthermore, it is recommended that the KDPS incorporate methods to facilitate prompt updates and model retraining, enabling the system to adjust swiftly to evolving market conditions. One potential approach is to implement periodic or event-driven updates, wherein the knowledge base and decision rules are revised in accordance with the most recent data and market conditions. By overcoming this constraint, the KDPS may offer more precise and pertinent observations, empowering real estate experts to make well-informed choices that accurately represent the present condition of the market.

8. The article lacks critical evaluation of the methodologies and findings of the referenced works. Each cited study is described briefly without assessing the strengths, weaknesses, or implications of the research. A critical evaluation would involve discussing the methodological rigor, applicability of findings, and potential biases in the studies cited.

Ans:

The strength and weakness of the work is included in the respective related works which helps to identify the implication of this work. The critical evaluation and finding of the work is included existing work.

Wang et al. [16] developed a DL model for house price prediction utilizing heterogeneous information analysis. The proposed model is a joint self-attention model that extracts necessary data from the database. The price prediction process evaluates the availability of amenities, rapid transit, location, and property environment. The proposed model identifies the important datasets that are required for prediction. The suggested model attains high accuracy in prediction, improving the systems' performance. However, the system requires additional effort to analyse a large data volume.

Chen et al. [17] introduced a semiconductor intellectual property (SIP) business model for value creation analysis. The key objective of the introduced model is to evaluate the actual value creation mechanism for SIP firms. The firms' intellectual property (IP) is identified, producing optimal data for business models. The introduced model classifies the IP based on specific characteristics and priorities. The SIP business model is mostly used for owners' entrepreneurship. The study offers insights into semiconductor intellectual property's economic models and dynamic capacities. However, its generalizability may be limited due to its special focus on the semiconductor industry. The generalizability of the findings and consequences to industries subject to distinct dynamics, technical landscapes, and intellectual property considerations may be limited.

Su et al. [18] proposed building information modelling (BIM) and machine learning (ML) integration framework automated property evaluation. An information extraction method is used here to identify important information from the database. ML mainly detects the key values required for the evaluation process. The suggested framework enhances the information exchange process among individuals. Numerical outcomes demonstrate that the proposed framework decreases the error ratio in the evaluation process. The potential amalgamation of Building Information Modeling (BIM) and machine learning methodologies for automated property valuation presents encouraging outcomes. However, it is important to acknowledge that the study's scope may be constrained by the accessibility and reliability of BIM data and the intricate nature of real-world situations that our models may not comprehensively encompass. Furthermore, the precision and dependability of the valuation models may be impacted by the particular datasets employed for training and validation.

Decision-Making Models for Realty Management

Hu and Lin [19] designed a multi-criteria group decision-making methodology (MCGDM) for property management systems under a z-number environment. The suggested technique is a novel aggregation method that decreases the complexity of management systems. MCGDM identifies the problems during decision-making that reduce the latency in the computation process. The suggested MCGDM increases the decision-making process's accuracy, enhancing the systems' performance range. The practical implementation of the proposed integrated multi-criteria group decision-making methodology for property concealment risk assessment may face challenges due to the intricate nature of collecting dependable and precise Z-number data, encompassing both fuzzy and probabilistic uncertainties. Furthermore, the efficacy of the methodology may be impacted by the proficiency and subjective assessments of the decision-makers engaged in the collective decision-making process.

Syed and Lawryshyn [20] introduced a multi-criteria decision-making technique for the risk analysis process. Important key values and Risk Matrix (RM) are identified from the database, producing appropriate data for further decision-making. A cost-benefit analysis is utilized here to analyze the appropriate data required for the systems. RM produces accurate datasets, which reduce the complexity and latency in the risk assessment process. The introduced method reduces the risk issues, increasing the network efficiency and effectiveness. Due to its complexity and processing overhead, the suggested multi-criteria decision-making approach may hinder its practical applicability in real-time or time-sensitive scenarios.

Fu and Hu [21] proposed a binary tree pricing method using a machine learning (ML) technique for farmland management systems. A strategy gradient reinforcement learning algorithm is implemented in the pricing technique to analyze the important features of the database. The proposed method uses basic principles which produce feasible conditions for the analysis process. Compared with other approaches, the proposed technique increases the financial criteria and performance of the networks. However the method consumes high computation complexity.

Lee et al. [22] introduced an adaptive decision-making method for civil infrastructure systems. The primary objective of the introduced technique is to evaluate the risks that occur during infrastructure systems. The introduced method identifies the dynamic changes in the communication and interaction process. Community risks and issues are identified, providing feasible data for decision-making. The introduced method achieves high accuracy in decision-making but consumes high computation time.

Anejionu et al. [23] developed a cloud-enabled big data infrastructure for spatial urban data systems (SUDS). The developed method uses an analytic technique that analyzes SUDS' important characteristics and features. Variations of urban keys and values are identified from the database, decreasing the computation process's latency. The real-time data source provides relevant data for the analytics process. Simulation outcomes illustrate that the suggested technique increases prediction accuracy, which improves SUDS's performance range. However, the method requires optimization approach while handling large volume of data.

Zhang and Carter [24] proposed a grazing system model for industries. The proposed model is an online information system that provides various services and functions to the users. Remote sensing techniques are used here to capture sensible data using wireless sensors. Wireless sensors reduce the difficulty of the computation process. The objective of the recommended model is to identify climate change in grazing lands. The recommended model enhances the overall effectiveness ratio of grazing land systems. Although, the training system requires additional effort to manage the scalability.

Zhang et al. [25] developed a data-driven decision strategy for offshore assets. A multicore classification model is utilized here that recognizes the exact information types based on the conditions. The data mining method is also used here to detect important datasets for decision-making. The multicore classification model improves offshore assets' feasibility and computation efficiency. The developed model maximizes the accuracy in decision-making, enhancing the systems' efficiency and reliability range.

März [26] introduced a new decision-making method for small private landlords (SPL). Both theoretical and practical aspects of the data, which produce relevant data for various processes, are identified. SPL gathers information from the low-demand housing market, providing feasible data for decision-making. Certain privacy policies and schemes are used in SPL, enhancing the systems' performance range. The presented method attains high accuracy in decision-making, high the computation process's complexity range.

Teresa [27] developed a racialized class-monopoly rent for land contracts. The primary objective of the proposed technique is to offer proper security services to the owners. The suggested technique identifies the vulnerabilities and issues that occur during rent. The identified effects provide relevant data for security policies. The proposed method creates various services via the class-monopoly process. The suggested approach maximizes the security and privacy range in the renting process. Although requires the standard security techniques to ensure the privacy on large volume of data.

9. While the article provides a comprehensive list of related works, it does not adequately discuss the relevance of each study to the proposed knowledge-based processing scheme (KDPS). The article should clearly articulate how each referenced work informs the development or validation of the KDPS and how it contributes to addressing the identified research problem. Discussing the relevance of previous studies would strengthen the rationale for the proposed approach and demonstrate its novelty or innovation.

Ans:

Highlighted the added portions that discuss the limitations of existing studies, such as industry specificity, data availability and quality issues, complexity in gathering reliable information, and computational overhead. Additionally, I have included a statement suggesting that the proposed work aims to address these limitations and research gaps by providing a more robust and widely applicable solution, employing advanced techniques to overcome data constraints and computational complexity challenges. The incorporation of domain-specific information and experience into the data analysis process is a limitation that is addressed by the knowledge-dependent data processing scheme (KDPS). The utilization of human expertise and decision-making rules allows the system to effectively manage limitations in data and surmount computational intricacies. The versatility of KDPS enables its application in several industries, effectively overcoming the challenge of limited generalizability through the integration of industry-specific knowledge and data sources.

10. One significant limitation is the scheme's reliance on historical data, which may not adequately capture real-time market dynamics. To address this, the KDPS should incorporate real-time data feeds and implement mechanisms for timely updates to adapt to changing market circumstances.

Ans:

It is recommended that the KDPS be improved by integrating real-time data streams from diverse sources, including property listings, market trends, and economic indicators. The constant integration of real-time data into the system enables the analytical and decision-making processes to be grounded in the most current information. Furthermore, it is recommended that the KDPS incorporate methods to facilitate prompt updates and model retraining, so enabling the system to swiftly adjust to evolving market conditions. One potential approach is to implement periodic or event-driven updates, wherein the knowledge base and decision rules are revised in accordance with the most recent data and market conditions. By overcoming this constraint, the KDPS may offer more precise and pertinent observations, empowering real estate experts to make well-informed choices that accurately represent the present condition of the market.

11. The effectiveness of the KDPS heavily relies on the quality and relevance of the features selected for analysis. There is a need to conduct thorough feature engineering and validation to ensure that the chosen features accurately reflect market trends and dynamics.

Ans:

Here the feature extraction is included in this process in which the efficiency of the system is evaluated using the success rate, data processing rate. These metrics are utilized to understand how effectively the extracted features involved in the realty management process.

12. Transparency and interpretability are crucial for building trust and understanding in AI-driven decision-making systems. The KDPS should provide clear visual representations of its decision-making processes to stakeholders, such as decision trees or decision borders, to enhance comprehension and trust.

Ans:

The KDPS has the capability to utilize methodologies such as decision tree visualization, which involves presenting the hierarchical arrangement of decision rules and their associated consequences in a user-friendly tree-like structure. Furthermore, the implementation of decision boundary visualizations allows for the graphical representation of decision borders or regions inside the feature space that correspond to various outcomes or recommendations generated by the KDPS.

13. Dealing with missing data is critical for developing robust data processing systems. The KDPS should implement robust strategies for handling missing values, such as imputation techniques, and conduct sensitivity analyses to evaluate the impact of different approaches on model performance.

Ans:

The KDPS method uses the stagnancy based data analysis that helps to handle the missing values in the dataset. The system uses equation 8 and 9 to explore the stagnancy examination which improves the overall robust of the study.

14. Optimal hyperparameter values significantly impact the performance of machine learning models. The KDPS should conduct thorough hyperparameter tuning studies to identify the best configuration for its algorithms and ensure optimal performance across different scenarios.

Ans:

**The hyperparameter tuning is performed to reduce the deviation between the outputs. varied hyperparameter values. Hyperparameter tuning studies aim to find the best values for the hyperparameters by methodically testing them. The recommendations are given to the upcoming process for successful sales values without any flaws; the process of verifying the procedure value with the market value. Here equation 10 and 11 utilized to examine the deviation between outputs according to the deviation, parameter tuning is performed. The detailed description is described in page no 12.**

15. Rigorous validation and evaluation processes are essential for assessing the KDPS's performance and reliability. Implementing techniques such as k-fold cross-validation can provide insights into the model's generalization performance and robustness.

**Ans:**

**The incorporation of a complete validation and evaluation methodology, such as k-fold cross-validation, is recommended for the KDPS. The process entails dividing the accessible data into k subsets, with one subset reserved for testing purposes, while the remaining k-1 subsets are utilized for training the model. The aforementioned procedure is iterated k times, wherein each subset is utilized as the test set. By conducting an assessment of the model's performance across various folds, a more dependable estimation of its capacity for generalization and resilience can be derived. Furthermore, it is imperative to conduct comprehensive testing of the KDPS on a wide range of real-world scenarios and extreme scenarios in order to evaluate its efficacy across different circumstances. This process entails the simulation of various market scenarios, property categories, and user specifications to ascertain the system's capacity to accommodate diverse inputs and deliver dependable suggestions or forecasts. From the given data, the requirement is extracted with failed maintenance, as presented in Fig. 6.**

16. Revise the manuscript to improve clarity by defining technical terms, providing detailed explanations of methodologies, and ensuring logical coherence between sections.

Ans:

**The detailed explanation and working process of proposed techniques, logical coherence between the sections are described clearly for better understandings. In addition, the technical terms are explained clearly.**

or flaws in the process. Identifying and correcting dataset outliers via effective outlier detection algorithms. Clustering, statistical approaches, or machine learning models developed for outlier identification may be used. Preprocessing the data using noise reduction methods helps lessen the effect of background noise and irrelevant data. The FCM algorithm clusters the data points based on fuzzy membership values, allowing each point to belong to multiple clusters with varying degrees of membership. It can effectively handle missing data by incorporating fuzzy membership values for missing features, enabling the imputation of missing values based on the cluster assignments

Dealing with missing data is essential to developing robust knowledge-dependent data processing systems (KDPS). If incorrectly handled, missing values in real-world datasets might affect how well models work. Focusing on how various imputation techniques affect KDPS performance using sensitivity studies. Choosing stable and reliable procedures after considering how different approaches to missing data management impact model predictions. The procedure of identifying the stagnancy based on the outcome is represented by the Equations (8) and (9) given below:

$\sum_{y\to\alpha} fN\left( V \right)=\tau(V)$	(8)

$\left. \begin{matrix} \sum_{y\to\alpha} Z_{F}=\int_{T_{Sh}}^{\alpha} \frac{1}{\xi}fI\left( \frac{r}{\xi} \right)dr \\ =1-F_{1}\left( \frac{T_{Sh}}{\xi} \right) \end{matrix} \right\}$

17. Provide a clear description of data processing and analysis techniques, including any software tools or algorithms utilized. Incorporate references to established methodologies to enhance transparency and reproducibility.

**Ans:**

**Clustering, statistical approaches, or machine learning models developed for outlier identification may be used. Preprocessing the data using noise reduction methods helps lessen the effect of background noise and irrelevant data. The FCM algorithm clusters the data points based on fuzzy membership values, allowing each data point to belong to multiple clusters with varying degrees of membership. It can effectively handle missing data by incorporating fuzzy membership values for missing features, enabling the imputation of missing values based on the cluster assignments. The relevant reference is placed in introduction and related work section.**

18. Conduct thorough validation of the proposed approach using benchmark datasets or simulated scenarios. Compare the performance of the proposed method with existing models through quantitative metrics and qualitative evaluation criteria.

**Ans:**

**The next section presents the comparative analysis using the metrics data processing rate, stagnancy detection, processing time, success rate, and training ratio. The variants are data inputs (20-260) and amenities (1-15). The additional methods considered are SMMP [25], BIM+AVM [18], and BTPM [21], discussed in the related works section.**

19. Simplify complex concepts and minimize technical jargon to make the manuscript accessible to a broader audience. Use clear, concise language and structured formatting to improve readability and comprehension.

**Ans:**

**Grammar correction and language corrections are corrected.**

20. While the conclusion briefly mentions the benefits of the proposed scheme, such as improved processing rate and success rate, it does not delve into the practical implications for real estate management practitioners or stakeholders.

**Ans:**

**Here, the sample scenario input is utilized to evaluate the described system efficiency. For the particular input, the system successfully works and ensure the particular success rate and processing rate. from the implications, the proposed system able to work with benchmark of data and real-time dataset.**

21. Discussing how the findings can inform decision-making processes, optimize resource allocation, or enhance overall operational efficiency in realty management would provide valuable insights for readers interested in applying the research in practical contexts.

**Ans:**

**The research conducted by KDPS has the potential to facilitate data-driven decision-making through the provision of comprehensive market insights, examination of patterns to identify risks and opportunities, and facilitation of scenario simulations. The optimization of resource allocation is achieved through the identification of areas that necessitate attention, the strategic targeting of marketing activities according to client preferences, and the alignment of workforce levels with workload projections. Operational efficiency is enhanced by implementing process automation, fostering cross-departmental collaboration through the utilization of integrated data perspectives, and enabling predictive maintenance through the analysis of data from multiple sources. In addition, KDPS improves client experiences by providing individualized recommendations, implementing focused marketing strategies, and addressing feedback. Furthermore, it supports compliance efforts and proactive risk mitigation methods.**

22. While the conclusion provides a concise summary of the research findings, it could benefit from greater specificity, depth, and discussion of practical implications. By enhancing the methodological explanation, providing illustrative examples, and discussing practical implications, the conclusion can better convey the significance and applicability of the research to readers.

**Ans:**

**The detailed summary and explanation, implication of this work is included in the conclusion section and highlighted.**

The research conducted by KDPS has the potential to facilitate data-driven decision-making by providing comprehensive market insights, examining patterns to identify risks and opportunities, and facilitating scenario simulations. Resource allocation optimisation is achieved by identifying areas that necessitate attention, strategically targeting marketing activities according to client preferences, and aligning workforce levels with workload projections. Operational efficiency is enhanced by implementing process automation, fostering cross-departmental collaboration through integrating data perspectives and enabling predictive maintenance through data analysis from multiple sources. In addition, KDPS improves client experiences by providing individualized recommendations, implementing focused marketing strategies, and addressing feedback. Furthermore, it supports compliance efforts and proactive risk mitigation methods.

Future research endeavours may prioritize advancing the KDPS by investigating sophisticated knowledge representation methodologies, such as deep learning-based approaches, to capture intricate patterns and correlations in the dataset effectively. Furthermore, the integration of real-time data feeds and the establishment of mechanisms for ongoing learning and adaptation would enhance the ability of the KDPS to deliver decision support that is both fast and accurate in the face of dynamic real estate market conditions

**Reviewer #3:**

Provide more information about the statistical approaches used in the data analysis, such as the models, tests, and significance levels, to help increase the results validity.

**Ans:**

**The detail validity of the work is described in section 4. In order to verify the validity and robustness of the data analysis, the KDPS utilizes a variety of statistical models and methodologies. Examples of regression models that can be employed to analyze the associations between different real estate variables and property values or market trends include linear regression, logistic regression, and ensemble approaches based on decision trees. In order to discover the most relevant models, many model selection strategies are utilized, including cross-validation and information criteria.**

Increase data accessibility by storing datasets in a public repository, making it easy for other researchers to validate and repeat the findings.

Ans:

**While this study utilizes a particular dataset for evaluating the efficiency of the system, future research will be enhanced by incorporating larger volumes of data, which can help improve the overall quality and robustness of the finding. Indicated that future research will incorporate larger and more diverse datasets. Highlighted that the inclusion of larger data volumes can potentially improve the overall quality and robustness of the research findings.**

Please clarify and improve the presentation of techniques, findings, and their significance to ensure improved comprehension and readability.

Ans:

**The presentation of this study is improved and technical corrections are performed which helps to improve the overall readability and comprehension of this work.**

Consider discussing the study's potential flaws, such as how successfully the proposed KDPS adapted to different real estate markets or situations, and how these flaws were resolved.

**Ans:**

**The research conducted by KDPS has the potential to facilitate data-driven decision-making through the provision of comprehensive market insights, examination of patterns to identify risks and opportunities, and facilitation of scenario simulations. The optimization of resource allocation is achieved through the identification of areas that necessitate attention, the strategic targeting of marketing activities according to client preferences, and the alignment of workforce levels with workload projections. Operational efficiency is enhanced by implementing process automation, fostering cross-departmental collaboration through the utilization of integrated data perspectives, and enabling predictive maintenance through the analysis of data from multiple sources. In addition, KDPS improves client experiences by providing individualized recommendations, implementing focused marketing strategies, and addressing feedback. Furthermore, it supports compliance efforts and proactive risk mitigation methods.**
